# Acute toxic encephalopathy induced by chloropicrin: case report

**DOI:** 10.3389/fmed.2026.1865213

**Published:** 2026-06-10

**Authors:** Luye Wang, Yingzhan Jiang, Weiwei Yan

**Affiliations:** Department of Critical Care Medicine, Weifang People’s Hospital, Shandong Second Medical University, Weifang, Shandong Province, China

**Keywords:** acute poisoning, chloropicrin, neurocritical care, toxic encephalopathy, trichloronitromethane

## Abstract

**Background:**

Chloropicrin is commonly utilized as a pesticide to fumigate soil against insects, fungi, and nematodes. As a highly toxic chemical substance, chloropicrin can cause irritation and toxic reactions at the site of contact. Current literature primarily focuses on reporting its toxic effects in human eyes, respiratory system and skin. Cases of toxic encephalopathy induced by chloropicrin remain exceedingly rare in the medical literature.

**Case presentation:**

A 53-year-old male developed impaired consciousness after exposure to chloropicrin, then he was admitted to the emergency department. After ruling out systemic diseases, cerebrovascular disorders, intracranial infections, and immune-mediated diseases, he was diagnosed with toxic encephalopathy induced by chloropicrin. Supportive treatment and the application of antioxidants and hyperbaric oxygen therapy were given to the patient. After 3 weeks, his consciousness improved, and he was discharged to home.

**Conclusion:**

We report a case of toxic encephalopathy induced by chloropicrin, and the patient only exhibited mild signs of acute respiratory distress syndrome (ARDS), which cannot explain his state of mental disorder. Although his head magnetic resonance imaging (MRI) was generally normal, the electroencephalography (EEG) supported the diagnosis of toxic encephalopathy. Through this case report and literature review, we aim to emphasize the need for enhanced protective measures among agricultural workers during production processes. Additionally, we seek to raise awareness among healthcare professionals regarding chloropicrin-induced toxic encephalopathy, advocating for timely diagnosis and effective management of poisoning exposures to improve patient outcomes.

## 1 Introduction

Toxic encephalopathy is a syndrome triggered by exposure to neurotoxic substances, characterized by neurological symptoms including headache, cognitive impairment, and altered consciousness. The primary pathological mechanisms involve capillary damage in the central nervous system, hypoxia, and cerebral edema (1).

Chloropicrin (trichloronitromethane, CCl_3_NO_2_) is a colorless liquid, which volatilizes slowly and can form toxic vapors such as phosgene, chlorine, and nitrogen oxides when released into the environment (2). Due to its high efficacy and relatively short half-life, it is commonly used as a fumigant in agricultural production for controlling insects, fungi, and nematodes. Chloropicrin soil fumigation not only effectively controls the occurrence of soilborne diseases but also significantly increases crop yield, and thus it is widely used in the cultivation of high-value crops such as ginger and strawberry (3–5). Management measures for chloropicrin include establishing buffer zones, mandating that handlers wear respirators when detected concentrations exceed action levels, and other protective strategies (6). Due to its volatility, inhalation is the main route of exposure. After contact, it will immediately irritate the eyes and respiratory tract, causing coughing and nausea in humans. Because of its small size and moderate lipophilicity, low concentration of chloropicrin can spread and damage medium and small bronchi whereas high concentration affects the entire respiratory tract, resulting in vomiting, dyspnea, and airway damage that may trigger bronchitis and pulmonary edema. Based on exposure duration, the estimated lethal dose for humans ranges from 120 to 300 ppm (7).

Chloropicrin is a highly toxic chemical substance that can induce irritation and toxic reactions at the contact site upon initial exposure. Due to its volatility, the eyes, skin, and respiratory system are direct targets of its effects. The relevant studies and literature reports mainly focus on its toxic effects in eyes (8–10), respiratory system (2, 11) and skin (12). No cases of toxic encephalopathy induced by chloropicrin have been reported to date, and its pathogenesis as well as management principles remain to be investigated.

## 2 Case presentation

A 53-year-old male, who was previously in good health, experienced dizziness, nausea and fatigue after fumigating the soil with chloropicrin while growing ginger. Two days later, he felt short of breath without fever. Three days later, he gradually developed consciousness disorders, presenting with unclear speech, irritability and shouting. He was sent to the local hospital for head CT scan and an MRI examination, but no cerebrovascular disease was found. After sedative treatment, no significant improvement was observed, and the patient was sent to the emergency department of our hospital. The neurological imaging examinations of the patients at admission are summarized in Figures 1, 2 shows the Chest X-ray at admission.

**Figure 1:**
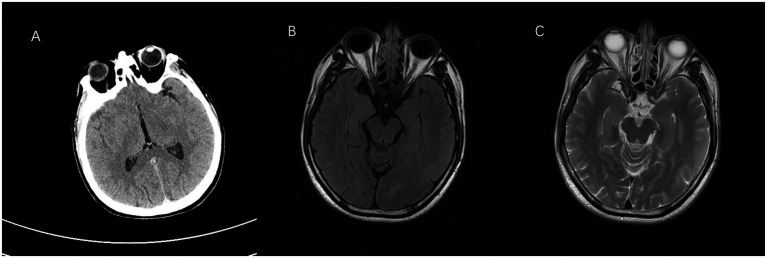
Neurological imaging examinations showed no significant abnormalities on admission. **(A)** Non-contrast head CT; **(B)** MRI T1WI; **(C)** MRI T2WI.

**Figure 2:**
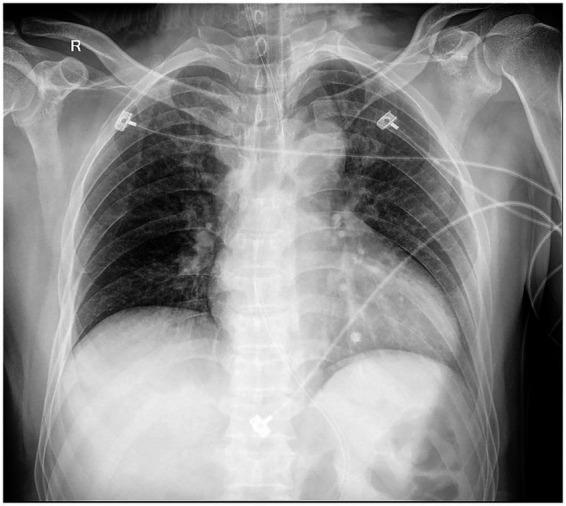
Chest X-ray showed no significant abnormalities on admission.

At admission, the patient was hemodynamically stable but exhibited extreme agitation and delirium (Richmond Agitation-Sedation Scale, RASS score 3–4), rendering standard Glasgow Coma Scale (GCS) assessment unfeasible. Neurological examination shows that both pupils have sensitive light reflex, the meningeal irritation sign is negative, no bilateral pathological signs are elicited, and the muscle tone of the limbs is normal. All other physical examinations showed no positive signs. After admission, given the patient’s disturbance of consciousness, invasive mechanical ventilation was initiated. Midazolam was used for sedation during mechanical ventilation, combined with ciprofol for the first 5 days. Sedative doses were titrated according to the RASS score, and a daily sedation interruption strategy was implemented to assess the patient’s neurological status. Lumbar puncture and cerebrospinal fluid analysis were performed. The cerebrospinal fluid pressure was 170cmH_2_O. The cerebrospinal fluid analysis showed that the number of nucleated cells was normal, with slightly elevated protein and glucose levels, and normal chloride ion levels. Bacterial culture, fungal culture, *Cryptococcus neoformans* smear, and acid-fast staining for *Mycobacterium tuberculosis* were all negative. The PCR tests for herpes simplex virus, cytomegalovirus and herpes simplex virus-2 were all negative. The metagenomic next-generation sequencing (mNGS) of cerebrospinal fluid was negative. Both autoimmune antibodies and paraneoplastic antibodies were negative. The concentration of chloropicrin in the blood was detected by mass spectrometry, and the result was negative. Three days later, a plain MRI scan of the head was re-examined and no obvious abnormalities were found. The result is shown in Figure 3. Fiberoptic bronchoscopy revealed that the airway was unobstructed, with no airway edema and no secretions. The patient was in good health in the past. There was no obvious evidence of hypoxia in the patient during the course of the disease. After admission, there was no evidence of functional damage to other organs in the laboratory examination. The imaging examination showed no obvious exudation signs in both lungs. The oxygenation index was 322 mmHg on admission, which fluctuated around 300 mmHg during hospitalization. Although there was a pulmonary infection, hypoxic–ischemic encephalopathy was not considered. After ruling out systemic diseases, encephalitis, meningitis, and rheumatic immune diseases, the patient had a clear history of exposure to chloropicrin, and was thus diagnosed with toxic encephalopathy. As there are currently no management guidelines for chloropicrin and its degradation products, we provide supportive treatment to the patient and apply antioxidants. The patient received intravenous administration of methylprednisolone, mannitol, sodium thiosulfate, reduced glutathione, and disodium adenosine triphosphate. After 2 weeks, the endotracheal tube was removed and hyperbaric oxygen therapy was initiated. After 3 weeks, the patient showed significant improvement in consciousness, GCS score E4V4M5, but with intermittent irritability, occasional self-talk, and sporadic irrelevant responses. EEG (Figure 4) revealed abundant slow waves without abnormal patterns. The patient was subsequently discharged to home. From the patient’s perspective, he complained of persistent auditory hallucinations during hospitalization, with a constant subjective perception that someone was talking to him. This neuropsychiatric symptom reflected his real-life experience and psychological distress caused by chloropicrin-induced toxic encephalopathy. Four weeks later, we conducted a telephone follow-up. The patient’s wife reported that the patient no longer exhibited irritability or delirious speech and could communicate normally with others. The clinical course of the patient was summarized in a timeline (Figure 5).

**Figure 3:**
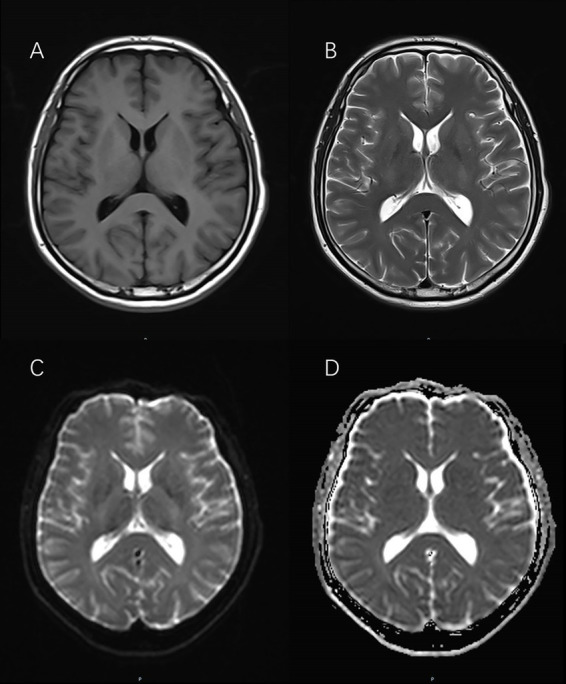
The ranial MRI examination showed no significant abnormalities 3 days after admission. **(A)** T1WI; **(B)** T2WI; **(C)** TRACE; **(D)** ADC.

**Figure 4:**
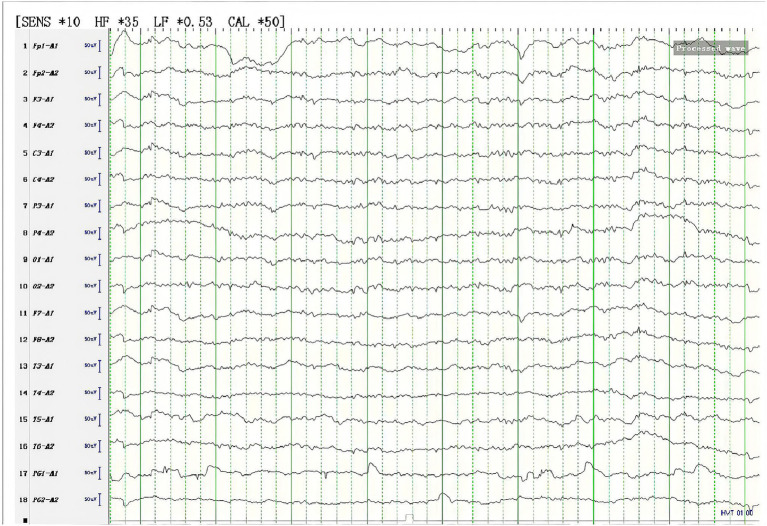
Electroencephalography revealed increased slow waves.

**Figure 5:**
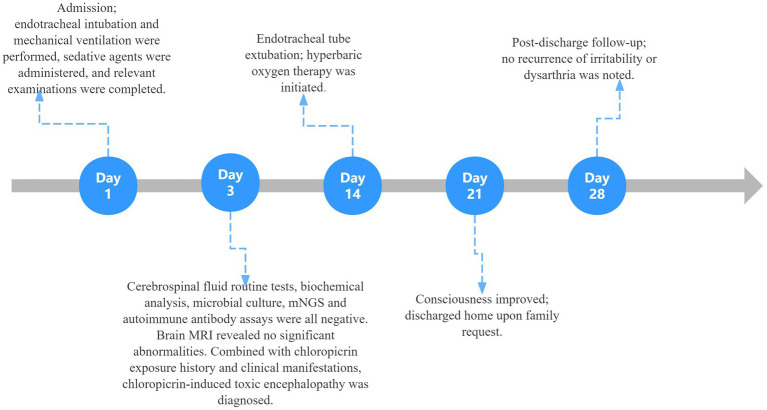
Timeline of clinical events in the patient with chloropicrin-induced toxic encephalopathy. Key interventions, diagnostic procedures, treatment adjustments, and follow-up outcomes are sequentially presented from Day 1 to Day 28.

The summary of laboratory and cerebrospinal fluid examination results on admission is presented in Tables 1–3.

**Table 1:** Laboratory examinations on admission.

Parameter	Value
Median blood counts	
Leukocytes, ×10^9^/L	19.39↑ (3.5–9.5)
Neutrophils, ×10^9^/L	18.19↑ (1.8–6.3)
Lymphocytes, ×10^9^/L	0.7↓ (1.1–3.2)
Hemoglobin, g/L	169 (130–175)
Platelets, ×10^9^/L	159 (125–350)
Main biochemical parameters	
Creatinine, μmol/L	94 (51–97)
Urea, mmol/L	7.2 (3.1–8.0)
C-reactive protein, mg/L	135↑ (<5.0)
PCT, mg/L	2.31↑ (<0.5)
Bilirubin, μmol/L	23.5 (≤26)
AST, U/L	18 (9–50)
ALT, U/L	25 (15–40)
LDH, U/L	307↑ (120–250)
Na, mmol/L	138 (137–147)

ALT, alanine aminotransferase; AST, aspartate aminotransferase; LDH, lactate dehydrogenase.

**Table 2:** Cerebrospinal fluid-related examinations.

Parameter	Value
Routine examination	
Characteristics	Colorless and transparent
Pressure, cmH_2_O	170 (70 ~ 180)
Red blood cell (RBC) count, ×10^12^/L	0
Nucleated cell count, ×10^9^/L	0.001
Qualitative pandy reaction	–
Biochemistry	
Glucose	5.43 ↑ (2.5–4.4)
Protein	415.2 ↑ (150–400)
Chloride	128.6 (120–132)
Microbiological examination	
Culture	—
Gram staining	—
Metagenomic next-generation sequencing	—

**Table 3:** Arterial blood gas analysis on admission.

Parameter	Value
pH	7.46↑ (7.35–7.45)
pCO2, mmHg	31.0↓ (35–45)
pO2, mmHg	129↑ (80–100)
Lac, mmol/L	1.4 (0.6–2)
HCO3-, mmol/L	22 (18–23)
BE(B), mmol/L	−0.9 (2–3)
SO2c, %	100 (95–98)
P/F, mmHg	322

## 3 Discussion

Chloropicrin is an inhalable toxic gas. Inhaling it can cause respiratory complications (2). Currently, there is no research data available on the distribution, metabolism, and excretion processes of chloropicrin in humans following exposure. The mechanisms underlying tissue damage induced by chloropicrin exposure remain unclear. The relevant studies and literature reports mainly focus on its toxic effects in eyes (8–10), respiratory system (2, 11) and skin (12). Animal research mainly focuses on eye injuries (13). There is no relevant research or case reports on the nervous system.

The mechanism of chloropicrin on respiratory epithelium includes increase the amounts of reactive oxygen species (ROS), activation of MAP-kinase (Erk1/2), and to elevate the expression of oxidative and endoplasmic reticulum stress (ER stress) associated proteins (14). In addition, research has found that chloropicrin can increase the expression of cell cycle regulatory proteins (p53, p27 and p21), trigger G2/M phase arrest, and significantly increase the number of autophagy/lysosomal vacuoles in human cell lines (7). The known mechanisms of pulmonary toxic reactions after exposure to bitter chloride include interfering with protein translation, ubiquitination modification and glycolysis processes, and inducing mitochondrial dysfunction (15).

Neurotoxins primarily affect gray matter structure, white matter, or both. As the most metabolically active tissues, gray matter is typically targeted by toxins that interfere with neuronal energy utilization (16). The potential mechanism by which chloropicrin induces toxic encephalopathy remains unexplored in current literature. Notably, The decomposition products of chloropicrin—chlorine (17) and phosgene (18)—can also cause central nervous system damage, complicating the understanding of its toxic encephalopathy mechanisms.

Although chloropicrin has adverse effects on human health, there are currently no specific analytical methods available for detecting chloropicrin exposure. The development of biomarkers faces challenges due to the rapid degradation of these compounds under physiological conditions. Given the inherent instability of chloropicrin, its detection in body fluids does not constitute an ideal biomarker (11).

No specific medical treatment regimen is recommended for chloropicrin and its degradation products, as chloropicrin and its metabolites can directly or indirectly activate multiple adverse pathways in the respiratory system. Therefore, a combination of therapeutic approaches may be required to mitigate oxidative damage, inhibit neuronal activation, reduce vascular permeability, and prevent secondary edema. Currently, there are no targeted treatments for chloropicrin-induced injuries. For exposed individuals, supportive therapy to maintain blood oxygen saturation remains the most critical intervention. Antioxidants have also been applied in the treatment of chloropicrin toxicity. N-acetylcysteine acts as a thiol-reducing agent to scavenge reactive oxygen species/reactive nitrogen species and stimulate glutathione synthesis and prevents chloropicrin-induced cytotoxicity and vacuolization in cell culture models (14).

The validity of our clinical findings is supported by comprehensive clinical evidence in this case. First, the patient had definite and direct chloropicrin exposure history. Second, his clinical manifestations, neuroimaging changes and electroencephalogram abnormalities were consistent with toxic encephalopathy induced by chloropicrin. Third, other common causes of acute encephalopathy were strictly excluded by laboratory and imaging examinations. Collectively, these data confirmed the causal relationship between chloropicrin intoxication and the patient’s neurological injury.

## 4 Conclusion

We report a case of toxic encephalopathy induced by chloropicrin, and the patient only exhibited mild signs of ARDS, which cannot explain his state of mental disorder. Although his head MRI was generally normal, the EEG revealed increased slow waves, and supported the diagnosis of toxic encephalopathy. Through this case, on one hand, it has enhanced our understanding of occupational-related health risks and underscored the urgency of implementing preventive measures during agricultural production to reduce exposure to harmful substances. On the other hand, this case report highlights the importance of early identification of neurotoxic substances in toxic encephalopathy, particularly in patients without significant medical histories. It serves as a reminder to healthcare providers to conduct thorough evaluations and detailed inquiries into medical histories to ensure timely diagnosis and effective management of toxic exposure, with the aim of improving patient outcomes.

## Data Availability

The original contributions presented in the study are included in the article/supplementary material, further inquiries can be directed to the corresponding author.

## References

[1] Dobbs MR. Toxic encephalopathy. Semin Neurol. (2011) 31:184–93. doi: 10.1055/s-0031-1277989 21590623

[2] Marzec J Nadadur S. Countermeasures against pulmonary threat agents. J Pharmacol Exp Ther. (2024) 388:560–7. doi: 10.1124/jpet.123.001822, 37863486 PMC10801713

[3] Sun Y Zeng R Fang W Hua J Huang S Wang Q . Mechanisms by which chloropicrin fumigation promotes soil potassium conversion and absorption. Front Microbiol. (2023) 14:1208973. doi: 10.3389/fmicb.2023.1208973, 37520378 PMC10373873

[4] Wang Y Yang X Xu M Geissen V. Effects of chloropicrin fumigation and Azoxystrobin application on ginger growth and phosphorus uptake. Ecotoxicol Environ Saf. (2022) 232:113246. doi: 10.1016/j.ecoenv.2022.113246, 35091296

[5] Baggio JS Cordova LG Toledo BF Noling JW Peres NA. A reassessment of the fungicidal efficacy of 1,3-Dichloropropene, chloropicrin, and Metam potassium against *Macrophomina phaseolina* in strawberry. Pest Manag Sci. (2022) 78:3416–23. doi: 10.1002/ps.6982, 35544358

[6] United States Environmental Protection Agency, Prevention P, Toxic S. Amended Reregistration Eligibility Decision (Red) for Chloropicrin. Washington, D.C.: U.S. Environmental Protection Agency, Prevention, Pesticides and Toxic Substances (2009).

[7] Pesonen M Storvik M Kokkola T Rysa J Vahakangas K Pasanen M. Transcriptomic analysis of human primary bronchial epithelial cells after chloropicrin treatment. Chem Res Toxicol. (2015) 28:1926–35. doi: 10.1021/acs.chemrestox.5b00123, 26352163

[8] Goswami DG Kant R Ammar DA Agarwal C Gomez J Agarwal R . Toxic consequences and oxidative protein Carbonylation from chloropicrin exposure in human corneal epithelial cells. Toxicol Lett. (2020) 322:1–11. doi: 10.1016/j.toxlet.2019.12.023, 31884112 PMC11249040

[9] Okoyeocha EOM Tewari-Singh N. Chloropicrin induced ocular injury: biomarkers, potential mechanisms, and treatments. Toxicol Lett. (2024) 396:70–80. doi: 10.1016/j.toxlet.2024.04.006, 38677567 PMC12239862

[10] Ruff AL Causey RD Koenig JA LaGrasta CV Gomez AM Chalmers BT . Sirna high throughput screening identifies regulators of chloropicrin and hydrogen fluoride injury in human corneal epithelial cell models. Exp Eye Res. (2022) 222:109169. doi: 10.1016/j.exer.2022.109169, 35820464

[11] Pesonen M Vahakangas K. Chloropicrin-induced toxicity in the respiratory system. Toxicol Lett. (2020) 323:10–8. doi: 10.1016/j.toxlet.2020.01.022, 31982502

[12] Gaskin S Heath L Pisaniello D Edwards JW Logan M Baxter C. Dermal absorption of fumigant gases during hazmat incident exposure scenarios-methyl bromide, Sulfuryl fluoride, and chloropicrin. Toxicol Ind Health. (2017) 33:547–54. doi: 10.1177/0748233716689651, 28196457

[13] Ebenezar OO Roney A Goswami DG Petrash JM Sledge D Komaromy AM . Ocular injury progression and cornea histopathology from chloropicrin vapor exposure: relevant clinical biomarkers in mice. Exp Eye Res. (2023) 230:109440. doi: 10.1016/j.exer.2023.109440, 36933694 PMC11658128

[14] Pesonen M Hakkinen M Rilla K Juvonen R Kuitunen T Pasanen M . Chloropicrin-induced toxic responses in human lung epithelial cells. Toxicol Lett. (2014) 226:236–44. doi: 10.1016/j.toxlet.2014.02.006, 24548678

[15] Pesonen M Rysa J Storvik M Rilla K Pasanen M Vahakangas K. Molecular targets of chloropicrin in human airway epithelial cells. Toxicol In Vitro. (2017) 42:247–54. doi: 10.1016/j.tiv.2017.05.003, 28476498

[16] Vonberg FW Blain PG. Neurotoxicology: a clinical systems-based review. Pract Neurol. (2024) 24:357–68. doi: 10.1136/pn-2023-003983, 38849205

[17] Achanta S Jordt SE. Toxic effects of chlorine gas and potential treatments: a literature review. Toxicol Mech Methods. (2021) 31:244–56. doi: 10.1080/15376516.2019.1669244, 31532270 PMC7108975

[18] Asgari A Parak M Nourian YH Ghanei M. Phosgene toxicity clinical manifestations and treatment: a systematic review. Cell J. (2024) 26:91–7. doi: 10.22074/cellj.2024.2011864.1405, 38459726 PMC10924841

